# Obstacles to Hygienic Achievement

**Published:** 1906-05-05

**Authors:** 


### OBSTACLES TO HYGIENIC ACHIEVEMENT.

In an address upon the above subject, Dr. William Butler [1] insists upon the vital importance of the public health, a platitude which has only lately won any recognition. Even now medicine is far from being considered a national concern, but is "indifferently sustained by the invalids on whom the State has thrown the onus of employing and maintaining the doctors." The author enumerates the most important causes operating to perpetuate disease among the population as follows: Callous indifference to the importance of healthy living, ignorant neglect of the simplest canons of health, wasteful and ill-directed economy in the household, intemperance, poverty, ineptitude and stupidity. The causes, therefore, are to be found more in the people themselves than in the virulence of the morbid processes which attack them. The growing prominence of these deficiencies is traced by Dr. Butler to the higher standard of culture, conduct, and hygiene, which is called for in order that the public health may remain good in spite of the progress of urbanisation which is so marked a feature of these days. Fifty years ago the urban and rural populations of England and Wales were practically equal. Twenty years later the urban population constituted 60 per cent. of the whole. By 1901 urban districts of not less than 10,000 inhabitants supplied a population twice as large as that of all smaller communities together. "The facts establish beyond dispute the truth that our population is becoming rapidly urbanised, so rapidly that in fifty years we have passed from being a semi-rural to an urban population, with an attenuating rural fragment destined soon to be, in respect of its size, a comparatively unimportant element in our society."

This immense wave of change has at last awaked a doubt in the public mind as to whether or not the national physique can tolerate and survive the altered conditions. The cry has been raised: "Back to the land." "But however desirable it may seem to get the people back to the land, it it to be feared that the problem is greater and more insoluble than that of making for them a healthy environment where they are." Dr. Butler considers that this latter task is not beyond the competence of society: that, given the essential condition of success—namely, a sane spirit of science—it is reasonable to believe that a population may be healthy though urbanised. But poverty so extreme as to preclude the provision of the bare necessities of life is, of course, fatal to health and efficiency, and such poverty, according to Mr. Chas. Booth and Mr. Rowntree, is the portion, at some period of their lives, of about one-third of our urban population. This period is that of the advent of the family, and the author suggests that public help should be extended to parents during this epoch in the family life.

A problem of public health which demands urgent consideration is the infantile mortality. It is imperative, urges the author, that women should be trained in the art of child-rearing. "It is little short of monstrous that a legalised union for purposes of procreation should be sanctioned by the State, when the prospective mother is at once so grossly ignorant and so little fitted by nature or education for the discharge of maternal function that it is a moral certainty that her offspring will perish of her unfittedness."

The remedy he suggests is the provision of a State Medical Service. There is, he says, no question that present methods of providing medical attendance for the masses have failed. But no remedy directed towards improvement in the people's opportunities of continuing healthy will touch the problem of the wastrel. "These parasitic dregs of our population, mentally and physically defective, congenital paupers and imbecile unemployables, preying on society, breeding and dying at a furious rate, but ever increasingly preserved at each step in our social progress: these unfortunate human pests surging in each centre of population, surviving where better types perish, crowding and burdening their more industrious neighbours, will batten, without benefiting, upon whatever provision is made for the social good. While insisting, on the one hand, on doing all that is possible to create an environment that shall give opportunity for health, such a view of the concrete conditions of human progress should be taken that, on the other hand, the means must be sought by which a period shall be put to the prolific multiplication of the undesirable dregs of our population."

[1] Public Health, Feb., 1906.

